# One health: Coming of age and antimicrobial resistance

**DOI:** 10.1017/S0950268824000529

**Published:** 2024-05-07

**Authors:** Dame Sally C. Davies

**Affiliations:** UK Special Envoy on Antimicrobial Resistance, Former Chief Medical Officer, England and Wales, Master of Trinity College, Cambridge, UK

2024 is an important year for moving forward both action and policy on antimicrobial resistance (AMR). There is the high-level meeting at the United Nations General Assembly, a meeting in Sweden of the UN Global Leaders Group on AMR and a Kingdom of Saudi Arabia Ministerial Meeting later in the year. All these meetings cry out for real-world data and evidence in order to develop the policies and actions we need across the globe. This excellent issue of Epidemiology and Infection will, I believe, help frame these discussions.

What do you think when you read or hear the term ‘one health’? For most people this is bewildering or unintelligible, for some an unnecessary complication of their work, and for politicians, it varies from meaning viruses that caused pandemics to discussions of trade barriers. Yet, reading this collection shows us the importance of considering the impact of working in one sector on other living animals and plants as well as on our planet. We know from climate studies that everything is interconnected. The exquisite balance of nature can be tipped all too easily into harming human health and food security and contaminating our environment.

This issue has ten papers reporting laboratory data on AMR that demonstrate that the prevalence of AMR now is significant and has been rising over time. The papers come from across the middle- and high-income countries showing us that AMR is not an isolated problem in any region. We misuse antibiotics and anti-infectives at our peril.

Previous data have generally shown higher levels of MRSA in pigs at slaughter than when sampled on farms. From the study of Smith et al. of MRSA in pigs slaughtered in England, we learn of the varied contamination levels across different abattoirs; this suggests cross-contamination between the lairage and point of post-stunning. They report that the slaughter processes have some effect in reducing contamination before the carcasses entered the chiller. Puangseree et al. from Thailand highlight the variety of plasmids as carriers of resistance genes in pigs, pork, and humans and how important it is that we investigate whether this circulation is due to horizontal transfer of plasmids or bacterial strain dissemination.

Four papers look at the prevalence of AMR in poultry. Rau et al. from Brazil showed high levels of resistance in salmonella species with multi-drug resistance found in 50.7% of isolates in 2014, rising to 77.3% in 2017. Mallioris et al. identified the main biosecurity practices associated with antimicrobial use (AMU) in European broiler farms to develop a statistical model that produces customized recommendations as to their best biosecurity measures.

In turkeys, Shrestha et al. assessed antimicrobial-resistant Campylobacter levels across Canada between 2016 and 2021 and found worryingly high levels. Importantly, they identified that a high proportion of C.jejuni and E.coli isolates were resistant to tetracycline and fluoroquinolones, despite the very low use of these antimicrobials in the turkey flocks studied. Canadian colleagues Phillips et al. undertook a helpful scoping review of factors potentially linked with AMR in turkeys and the 13 references included in this study reported 36 factors of which AMU and the potential associations with AMR are most frequently cited.

Two scoping reviews, also from Canada, looked at Campylobacter. Isada et al. characterized the burden of illness measures associated with human quinone-resistant Campylobacter species infections as these are one of the most common causes of bacterial gastroenteritis worldwide. The 26 studies they found yielded mixed results on burden of infection, highlighting how difficult it is to assess the magnitude of the problem at this time. Neustaedter et al. included 27 articles in their review also showing how difficult it is to identify consistent risk factors because of the heterogenicity of results, inconsistent analysis, and lack of data from low- and middle-income countries. They did show that important factors linked to an increased risk of infection with fluoroquinolone-resistant strains include foreign travel and prior AMU.

Two further studies from Canada address AMR in shrimp and salmon, using the Codex Guidelines for Risk Analysis of Foodborne AMR. Young et al. undertook a scoping review of the distribution and frequency of extended-spectrum beta-lactamase (EBSL)-producing Enterobacteriaceae, finding 16 relevant studies, while Loest et al. helpfully looked at the risk profile, using the Codex Framework, addressing carbapenem-resistant *E. coli.* They describe and define the food/bacteria/antimicrobial combination to guide decision-makers towards the next steps in risk analysis procedure. These studies demonstrate that the Codex Guidelines can be applied not only to terrestrial animals, but also to non-terrestrial food animals species, providing a transparent and structured format for inclusion of additional considerations of the water environment.

Primeau et al. also investigated ESBLs using data from the excellent Canadian Integrated Program for Antimicrobial Resistance Surveillance (CIPARS), highlighting the relatively low prevalence of potential EBSL-EC and EBSL-SA in animals, food, and humans across Canada. The total number fell over the study from 2012 to 2017, though this was not a statistically significant trend. The fall is ascribed to the 2014 poultry industry initiative that banned the preventative use of ceftiofur in poultry.

This series is truly one Health with the study from Singh et al. looking at the public health implications of plasmid-mediated quinone and aminoglycoside resistance genes in *E. coli* inhabiting a major anthropogenic urban river of Northern India. This matters as *E. coli* are a major reserve of AMR genes that can be disseminated to other bacteria by horizontal gene transfer. Indeed, all the AMR genes discerned in the aquatic *E. coli* were found to be situated on conjunctive plasmids, and thus easily transferable.

Fasti et al. explored animal sources of AMR infections in humans through a systematic review, identifying 31 relevant publications, including 12 risk assessments. As across the whole of this Epidemiology and Infection AMR special issue, the most frequently addressed animal sources were poultry, followed by cattle and pigs. We cannot escape the urgent need for better research on causation. Studies on the direct contribution of animal sources of AMR remain too rare although helpfully, we are seeing increasing data on this association.

A study from 18 children’s Day-care Centres in Belgium addresses the epidemiology of multi-drug resistant bacteria using molecular typing across three winter months in 2018 and 2019. The EBSL Enterobacterales prevalence was high at 15.8%, and varied across facilities, re-emphasizing the central role of hygiene and infection control measures in prevention.

Fungi have not been ignored in this issue with two studies: in the first from a South China Hospital of Aspergillosis cases, Bilal et al. reported the rising levels over the five years studied with the highest prevalence seen in patients from the Intensive Care Unit. A rising level of resistance to triazole treatments is reported and put down to their extensive use in agriculture, which accounts for over one-third of all antifungal use in China. The second study investigated invasive candidiasis in a University Hospital in Thailand before and during COVID-19. Szekely et al. show that resistance to treatment is rising so empirical treatment is now inappropriate.

These studies are all-important as they show what can be done and demonstrate convincingly our need for routine linked surveillance between animals and humans, the food chain, and human health services. If we are to protect modern medicine and our food chain, such connected data will be essential.

The final paper in this series, from Sutton and Ashley, gives an excellent summary of why and how antibiotics have been misused ever since their discovery, leading to antibiotic resistance (AMR) and its consequences on human lives and health service costs. Antibiotics are global goods that underpin modern health systems. We need, through stewardship, to conserve the antibiotics we have for both humans and animals. We also badly need innovation in new antibiotics, treatments, and diagnostics. Sutton and Ashby give sound advice on the actions needed to maintain our health systems.

I hope that countries do come together and agree actions at the UNGA High Level Meeting on AMR to improve our connected surveillance, to improve antimicrobial stewardship and appropriate use, to establish an Independent Panel to assess the evidence and advise on targets and policies, while improving access for all to both old antibiotics and innovation. Time is running out, but it is not too late to act on AMR.

